# The Role of Emotions in Health Literacy and Patient Education: Trends, Technologies, and Future Opportunities—A Scoping Review

**DOI:** 10.3390/healthcare14152424

**Published:** 2026-08-06

**Authors:** Monica Daniela Gómez-Rios, Miguel Angel Quiroz-Martinez, Santiago Castro Arias, María Kourilovitch

**Affiliations:** 1 Carrera de Computación, Universidad Politécnica Salesiana, Sede Guayaquil, Guayaquil 090109, Ecuador; mquiroz@ups.edu.ec (M.A.Q.-M.); scastroa1@ups.edu.ec (S.C.A.); 2 UNERA (Unit of Rheumatic Disease and Autoimmunity), Hospital Monte Sinaí, Cuenca 010107, Ecuador; maria.kourilovitch@uneracuenca.com

**Keywords:** health literacy, patient education, emotions, emotional well-being, anxiety, emotion assessment, artificial intelligence, emotion recognition, affective computing, augmented reality, digital health, scoping review

## Abstract

**Highlights:**

**What are the main findings?**

- Anxiety and emotional distress were the most frequently investigated emotional outcomes in health literacy and patient education studies.
- Educational programs and educational materials were the most commonly used approaches, whereas artificial intelligence and emotion recognition technologies were rarely incorporated.

**What are the implications of the main findings?**

- Emotions influence key patient education outcomes, particularly understanding, emotional well-being, adherence, and communication.
- Integrating artificial intelligence, emotion recognition, and augmented reality may support the development of more personalized, adaptive, and emotionally responsive patient education interventions.

**Abstract:**

Background/Objectives: Health literacy and patient education play a key role in supporting informed decision-making, treatment adherence, and disease self-management. While educational interventions have traditionally focused on improving knowledge and understanding, emotions are increasingly recognized as factors associated with learning processes and healthcare experiences. This scoping review mapped the role of emotions in health literacy and patient education by identifying the emotions investigated, assessment methods, technologies employed, educational outcomes, and opportunities for emerging technologies. Methods: A scoping review was conducted following PRISMA-ScR and Joanna Briggs Institute guidance. Searches were finalized in Scopus and PubMed on 6 June 2026, with no publication-date restrictions; only English-language journal articles and reviews were eligible. Two reviewers screened records, extracted data, and classified studies, resolving disagreements by consensus and consulting methodological or clinical co-authors when needed. Results: A total of 135 studies were included. Anxiety and emotional distress were the most frequently investigated emotional categories, whereas emotional support, reassurance, and emotional engagement received less attention. Interviews, surveys, questionnaires, and standardized scales were the predominant assessment methods. Emotional well-being and understanding were the most frequently reported educational outcomes, followed by adherence, communication, and emotional support. Educational programs and educational materials were the most commonly employed approaches, while digital health solutions appeared less frequently. Limited use of artificial intelligence and objective emotion-recognition methods was identified. Conclusions: Emotional factors were associated with several patient-education outcomes, but the heterogeneous and predominantly self-reported evidence does not establish causality. Emerging technologies warrant further evaluation before their effectiveness, feasibility, and safety in patient education can be established.

## 1. Introduction

Health literacy has become an essential component of modern healthcare systems because it influences individuals’ ability to access, understand, evaluate, and apply health-related information in ways that support informed decision-making and effective disease management. Adequate health literacy has been associated with improved health outcomes, greater treatment adherence, enhanced self-management skills, and more active participation in healthcare processes [1,2]. Consequently, patient education has emerged as a fundamental strategy for promoting health literacy by providing individuals with the knowledge and skills necessary to understand medical information and make informed decisions regarding their health.

For the purposes of this review, emotion is understood as a time-limited, context-dependent response involving subjective experience together with cognitive appraisal, behavioral expression, motivational tendencies, and physiological change. This distinction is important because the included literature uses related but non-equivalent constructs, including anxiety, fear, distress, reassurance, emotional support, engagement, and well-being. Health literacy concerns patients’ capacity to access, understand, appraise, and use health information, whereas patient education comprises the planned activities through which such capacities may be supported. Emotional responses can shape attention, information processing, communication, and willingness to act on health information; conversely, comprehensible and supportive education may reduce uncertainty and emotional burden. This reciprocal relationship provides the conceptual basis for mapping emotions across health-literacy and patient-education studies.

Patient education encompasses a wide range of interventions, including educational programs, counseling sessions, printed materials, audiovisual resources, and digital health technologies. Traditionally, these interventions have focused on improving patients’ knowledge and comprehension of health information. However, educational effectiveness depends not only on cognitive factors but also on emotional experiences that influence how information is received, interpreted, and applied. Patients often encounter emotional challenges throughout healthcare processes, and these emotions may shape their engagement with educational materials, communication with healthcare professionals, and adherence to recommended treatments [3,4].

Emotions such as anxiety, fear, distress, uncertainty, reassurance, confidence, and emotional support frequently accompany healthcare experiences. These emotional responses can either facilitate or hinder learning and decision-making processes. Negative emotions may reduce attention, impair information processing, and increase avoidance behaviors, whereas positive emotional experiences can improve engagement, confidence, coping, and understanding. Consequently, emotions have increasingly been recognized as relevant factors influencing the success of patient education interventions and the development of health literacy [1,3].

At the same time, technological advances have expanded the possibilities for supporting patient education. Digital health solutions, mobile applications, web-based educational platforms, and other technology-enhanced interventions provide new mechanisms for delivering information and supporting patients throughout their healthcare journeys. More recently, developments in artificial intelligence have introduced opportunities for analyzing patient behaviors and personalizing educational experiences. Technologies capable of recognizing emotional states through facial expressions, speech patterns, physiological signals, or behavioral indicators have attracted growing interest due to their potential to support more adaptive and responsive educational environments [2,5]. Similarly, augmented reality has demonstrated potential to improve patient understanding, engagement, and satisfaction through interactive educational experiences [6,7].

Despite increasing recognition of the importance of emotions in healthcare, the existing literature remains fragmented across different health conditions, educational approaches, assessment methods, and technological contexts. Studies have examined a variety of emotional factors and educational interventions, yet there is limited synthesis of how emotions are addressed within the broader fields of health literacy and patient education. Furthermore, the potential role of emerging technologies, including artificial intelligence, emotion recognition, and immersive educational environments, has not been comprehensively examined from the perspective of patient education.

Therefore, this scoping review aims to analyze the role of emotions in health literacy and patient education by identifying the emotions investigated, the methods used to assess them, the technologies employed to support patient education, the influence of emotions on educational outcomes, and future opportunities for emerging technologies.

To achieve this objective, the review addresses the following research questions:

RQ1. What emotions have been studied in health literacy and patient education?

RQ2. How are these emotions assessed?

RQ3. How do emotions influence understanding, adherence, and decision-making?

RQ4. What technologies have been used to support patient education while considering emotional factors?

RQ5. What opportunities exist for emerging technologies such as artificial intelligence, emotion recognition, and augmented reality in patient education?

By synthesizing evidence from studies conducted across diverse healthcare contexts, this review provides an overview of current research trends, identifies gaps in the existing literature, and highlights future directions for the development of more effective, personalized, and emotionally responsive patient education strategies.

## 2. Materials and Methods

This section describes the methodological procedures used to identify, select, analyze, and synthesize the available evidence regarding the role of emotions in health literacy and patient education. The review process included study identification, screening, data extraction, and thematic analysis to address the research questions and map the current state of the literature.

### 2.1. Study Design

This study was conducted as a scoping review to map and synthesize the existing literature on the role of emotions in health literacy and patient education. The methodological approach was informed by the framework proposed by [8] and later refinements by [9]. The review followed the Preferred Reporting Items for Systematic Reviews and Meta-Analyses Extension for Scoping Reviews (PRISMA-ScR) guidelines [10] and considered the updated methodological guidance provided by the Joanna Briggs Institute [11].

No formal review protocol was prepared or registered before the review. Nevertheless, the review procedures were prospectively structured around the review questions, eligibility criteria, extraction variables, PRISMA-ScR reporting framework, and Joanna Briggs Institute methodological guidance.

### 2.2. Search Strategy

A literature search was conducted in Scopus and PubMed, and the final database search was completed on 6 June 2026. No publication-date restrictions were applied, and only studies published in English were eligible. Scopus was selected for its multidisciplinary coverage of health, education, social science, and technology literature, whereas PubMed was selected for its biomedical and clinical coverage. The use of two databases was a pragmatic decision aligned with the interdisciplinary scope of the review; however, studies indexed exclusively in other databases may have been missed. Reference lists of included articles were not systematically searched for additional records.

(“patient education” OR “health education”) AND (“health literacy”) AND (emotion* OR emotional) AND patient* AND NOT (“mental health” OR depression OR schizophrenia OR psychosis OR autism OR eating disorder*) AND NOT (student* OR undergraduate* OR university OR “medical education” OR “nursing education”)

The search focused on patient populations and educational interventions. The NOT operators were used to preserve the review’s predefined scope by excluding records centered primarily on mental-health disorders, university students, medical or nursing education, and healthcare-professional training. Because exclusion operators can remove potentially relevant records when terminology overlaps, titles and abstracts of retrieved records were screened against the eligibility criteria, and the possible loss of relevant evidence is acknowledged as a limitation. All the retrieved records were exported for deduplication and screening.

### 2.3. Eligibility Criteria

The eligibility criteria used during the screening process are presented in Table 1.

### 2.4. Study Selection

The retrieved records were screened in multiple stages by two reviewers (M.G.R. and S.C.). First, records not meeting the document-type requirements were removed. Titles and abstracts of the remaining records were then evaluated against the predefined eligibility criteria. Disagreements were discussed until consensus was reached. When uncertainty persisted, M.Q. was consulted for methodological issues and M.K. for clinically focused questions.

**Figure 1:**
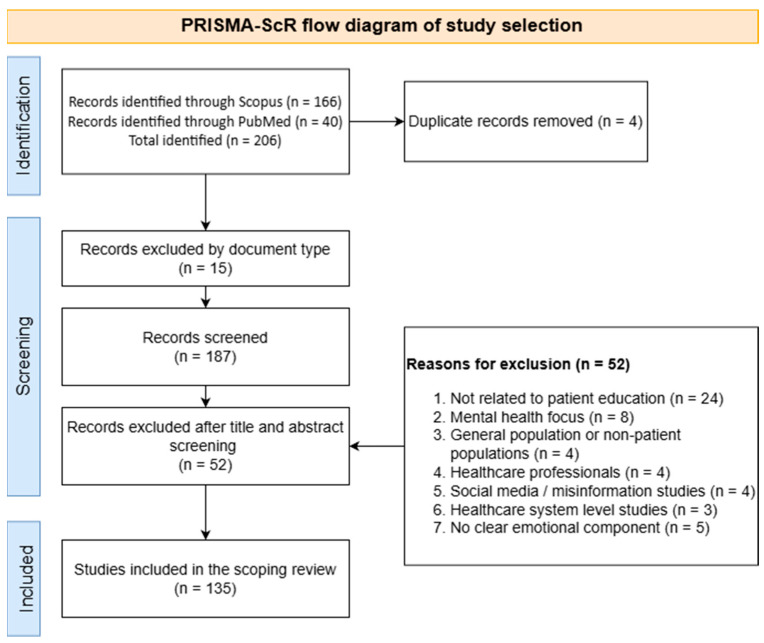
PRISMA-ScR flow diagram of study selection.

The screening process focused on identifying studies addressing emotions within patient education and health literacy contexts while excluding records outside the scope of the review. The complete study selection process is presented in the PRISMA-ScR flow diagram shown in Figure 1.

### 2.5. Data Extraction

Data were extracted by two reviewers (M.G.R. and S.C.) using a structured matrix developed for this review. The matrix was initially applied to a small subset of studies and refined iteratively to ensure consistency in the extracted variables, thematic categories, and outcome classifications before being applied to all the included studies. Extracted information included bibliographic characteristics, health condition, educational context, emotions addressed, assessment methods, technologies employed, and principal findings. For studies classified in the Emotional Response category, educational outcomes associated with emotions were also recorded. Discrepancies were resolved through discussion and consensus, with methodological or clinical consultation when required.

### 2.6. Data Analysis

The analysis was conducted in two stages. First, the included studies were classified into thematic categories according to their predominant objective and analytical focus. The categories were developed iteratively and included Health Literacy, Emotional Response, Digital Health, Patient Empowerment, Self-Management, and Artificial Intelligence. Although some studies addressed more than one dimension, each study was assigned to one primary category to avoid double counting. M.G.R. and S.C. performed the classification and resolved uncertain cases by consensus, consulting M.Q. or M.K. when methodological or clinical interpretation was required. Frequencies and percentages were calculated to describe the distribution of studies across categories.

Second, studies classified within the Emotional Response category were subjected to a more detailed analysis. This analysis examined the emotions investigated, technologies employed, health conditions addressed, assessment methods used, and educational outcomes associated with emotional factors. Descriptive statistics were used to summarize the extracted information and identify the most common patterns reported in the literature.

Given the scoping-review design and the substantial heterogeneity in study populations, health conditions, educational interventions, emotional constructs, assessment instruments, and reported outcomes, no inferential statistical analysis or meta-analysis was conducted. The analysis was intended to map the distribution of the evidence and identify recurring research patterns rather than to estimate pooled intervention effects.

## 3. Results

This section presents the findings of the scoping review, including the study selection process, thematic classification of the included studies, emotional outcomes investigated, technologies employed, health conditions addressed, assessment methods, and the reported influence of emotions on patient education outcomes. Study-level characteristics of the included records are summarized in the supplementary tables, whereas the main text highlights the patterns that answer the research questions.

### 3.1. Study Selection

A total of 206 records were identified through database searching, including 166 records from Scopus and 40 records from PubMed. After removing 4 duplicate records and excluding 15 records based on document type, 187 records remained for title and abstract screening. Title and abstract screening resulted in the exclusion of 52 records that did not meet the predefined eligibility criteria. Consequently, 135 studies were included in the scoping review (Figure 1).

The 52 records excluded after title and abstract screening were primarily removed because patient education was not the main focus of the study (*n* = 24). Additional exclusions included studies focused on mental health (*n* = 8), general or non-patient populations (*n* = 4), healthcare professionals (*n* = 4), social media or misinformation studies (*n* = 4), healthcare system-level studies (*n* = 3), and studies lacking a clear emotional component (*n* = 5).

### 3.2. Thematic Classification of Included Studies

To provide an overview of the research landscape, the included studies were classified into thematic categories according to their primary focus. This classification included Health Literacy, Emotional Response, Digital Health, Patient Empowerment, Self-Management, and Artificial Intelligence. The distribution of studies across these categories is presented in Table 2.

The 135 included studies were classified into six thematic categories according to their primary focus [12,13,14,15,16,17,18,19,20,21,22,23,24,25,26,27,28,29,30,31,32,33,34,35,36,37,38,39,40,41,42,43,44,45,46,47,48,49,50,51,52,53,54,55,56,57,58,59,60,61,62,63,64,65,66,67,68,69,70,71,72,73,74,75,76,77,78,79,80,81,82,83,84,85,86,87,88,89,90,91,92,93,94,95,96,97,98,99,100,101,102,103,104,105,106,107,108,109,110,111,112,113,114,115,116,117,118,119,120,121,122,123,124,125,126,127,128,129,130,131,132,133,134,135,136,137,138,139,140,141,142,143,144,145,146]. Health Literacy accounted for 27.4% of the studies (*n* = 37), followed by Emotional Response with 26.7% (*n* = 36). Digital Health represented 17.0% of the studies (*n* = 23), while Patient Empowerment accounted for 16.3% (*n* = 22). Self-Management represented 8.9% of the studies (*n* = 12), and Artificial Intelligence accounted for 3.7% (*n* = 5).

Detailed study-level characteristics of all 135 included studies, including author and year, country, study objective, methodological design, population or sample, healthcare context, and main findings relevant to the review questions, are provided in Supplementary Table S1. Supplementary Table S2 provides the detailed characteristics of the 36 studies classified within the Emotional Response category [111,112,113,114,115,116,117,118,119,120,121,122,123,124,125,126,127,128,129,130,131,132,133,134,135,136,137,138,139,140,141,142,143,144,145,146].

The 36 studies analyzed in Section 3.3, Section 3.4, Section 3.5, Section 3.6 and Section 3.7 represent the Emotional Response subset, not the complete evidence base. The broader thematic map and conclusions concerning the research landscape are based on all 135 included studies, while the more detailed emotion, instrument, technology, condition, and outcome analyses are restricted to this predefined subset.

### 3.3. Emotional Outcomes

Among the 36 studies classified within the Emotional Response category, anxiety was the most frequently assigned primary emotional category (30.6%, *n* = 11), followed by emotional distress (27.8%, *n* = 10). Fear, emotional support, and emotional perception each accounted for 8.3% (*n* = 3), while emotional engagement accounted for 5.6% (*n* = 2). Four less frequently reported categories—emotional well-being, reassurance, stigma, and a nonspecific emotional response—were each identified in one study and together represented 11.1% (*n* = 4). Each study was assigned to one primary emotional category for this descriptive analysis.

Table 3 shows that anxiety and emotional distress were the most frequently examined emotional outcomes within the Emotional Response subset. Anxiety was identified in 11 studies (30.6%) across surgical, oncological, respiratory, renal, and chronic disease contexts. These studies linked lower health literacy, information overload, uncertainty, and unclear communication with greater anxiety. In contrast, tailored explanations, structured education, and empathetic communication were generally associated with improved understanding and lower emotional burden [113,114,118,122,124,125,127,132,133,140,145].

Emotional distress was identified in 10 studies (27.8%), mainly in cancer, diabetes, HIV/AIDS, screening, and bad-news communication contexts. Distress was commonly associated with limited understanding, treatment burden, negative educational experiences, and communication difficulties. Personalized education, psychosocial support, and clearer communication helped patients cope more effectively and participate more actively in their care [111,115,117,128,131,139,141,143,144,146].

Fear, emotional support, and emotional perception each accounted for three studies (8.3%). Fear was particularly evident in situations involving serious diagnoses, transplantation, and complex treatment decisions [112,120,137]. Emotional support from clinicians, relatives, and peers facilitated coping, access to care, and decision-making [119,126,136]. Studies addressing emotional perception showed that patients’ beliefs and emotional interpretations of illness and treatment influenced adherence, risk perception, and understanding [134,135,138].

Emotional engagement was reported in two studies (5.6%), both of which used narrative or book-based educational strategies. These approaches increased engagement and were associated with stronger dialogue, adherence, and intentions to adopt healthier behaviors [129,130].

Four additional studies (11.1%) addressed less frequently reported outcomes, including emotional well-being, reassurance, stigma reduction, and general emotional response. These findings suggest that concise, culturally tailored, and developmentally appropriate materials can strengthen confidence, coping, and emotional adaptation [116,121,123,142].

**Figure 2:**
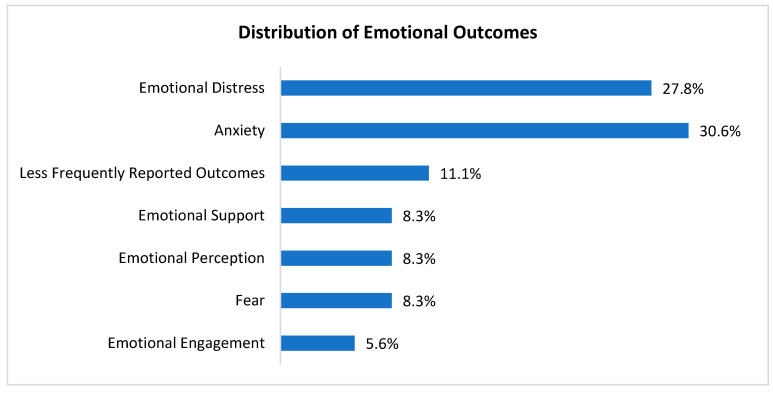
Distribution of emotional outcomes in Emotional Response studies (*n* = 36). Less frequently reported outcomes included emotional well-being, reassurance, stigma, and emotional response, each identified in a single study.

Based on this classification, Figure 2 presents the distribution of the primary emotional outcomes identified across the 36 studies.

Anxiety was the most frequently reported primary emotional category (30.6%, *n* = 11), followed by emotional distress (27.8%, *n* = 10). Fear, emotional support, and emotional perception each represented 8.3% (*n* = 3), and emotional engagement represented 5.6% (*n* = 2). The four less frequently reported categories collectively accounted for 11.1% (*n* = 4).

### 3.4. Technologies Used

**Figure 3:**
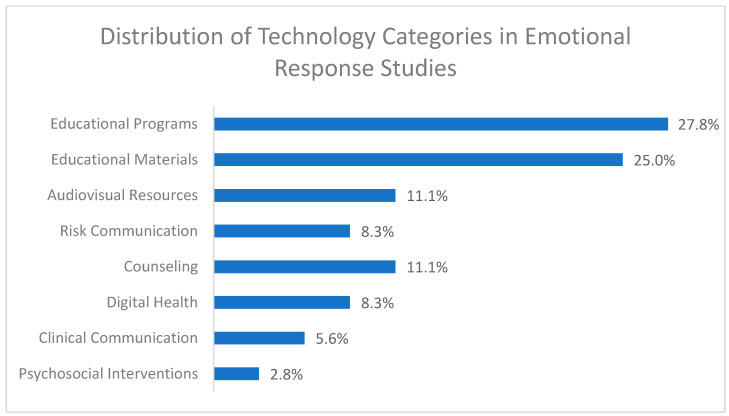
Distribution of educational, communication, and technology-based approaches identified in Emotional Response studies (*n* = 36).

The reviewed studies employed a variety of educational, communication, and technology-based approaches. The identified categories included educational programs, educational materials, audiovisual resources, counseling, risk communication, digital health, clinical communication, and psychosocial interventions. The distribution of technology categories identified within the Emotional Response studies is presented in Figure 3.

Educational programs accounted for 27.8% of the studies (*n* = 10), followed by educational materials with 25.0% (*n* = 9). Audiovisual resources and counseling each represented 11.1% of the studies (*n* = 4). Risk communication and digital health accounted for 8.3% of the studies (*n* = 3) each. Clinical communication represented 5.6% (*n* = 2), while psychosocial interventions accounted for 2.8% of the studies (*n* = 1).

### 3.5. Health Conditions Addressed

**Figure 4:**
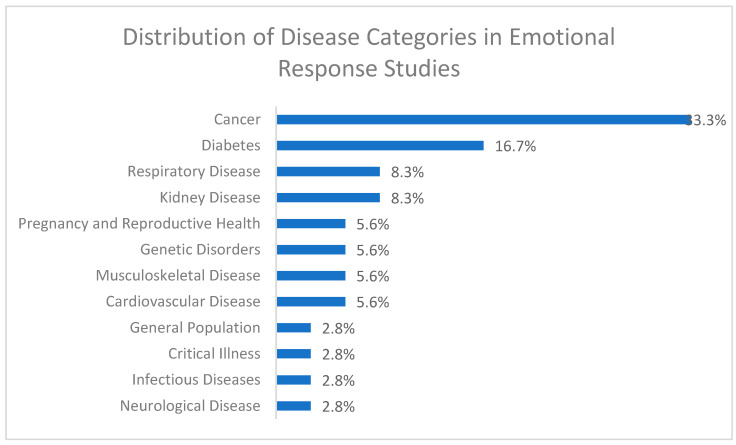
Distribution of health conditions addressed in Emotional Response studies (*n* = 36).

The Emotional Response studies addressed a range of health conditions, including cancer, diabetes, respiratory disease, kidney disease, musculoskeletal disease, cardiovascular disease, genetic disorders, pregnancy and reproductive health, neurological disease, infectious diseases, critical illness, and studies involving the general population. The distribution of disease categories is presented in Figure 4.

Cancer accounted for 33.3% of the studies (*n* = 12), followed by diabetes with 16.7% (*n* = 6). Respiratory disease and kidney disease each represented 8.3% of the studies (*n* = 3). Cardiovascular disease, musculoskeletal disease, genetic disorders, and pregnancy and reproductive health each accounted for 5.6% (*n* = 2). General population, critical illness, infectious diseases, and neurological disease each represented 2.8% of the studies (*n* = 1).

### 3.6. Instruments Used to Assess Emotions

**Figure 5:**
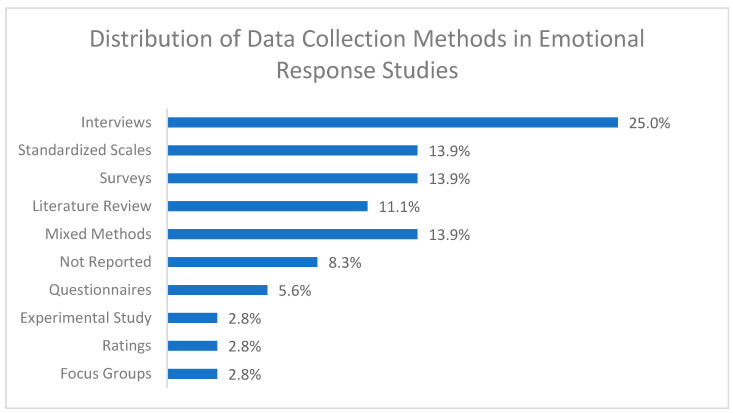
Distribution of data collection methods used in Emotional Response studies (*n* = 36).

A variety of instruments were used to assess emotional aspects within patient education contexts. The identified data collection methods included interviews, standardized scales, surveys, mixed methods, literature reviews, questionnaires, experimental studies, ratings, focus groups, and studies in which the instrument was not reported. The distribution of data collection methods is presented in Figure 5.

Interviews accounted for 25.0% of the studies (*n* = 9). Standardized scales, surveys, and mixed methods each represented 13.9% of the studies (*n* = 5). Literature reviews accounted for 11.1% (*n* = 4). Studies in which the instrument was not reported represented 8.3% (*n* = 3), while questionnaires accounted for 5.6% (*n* = 2). Experimental studies, ratings, and focus groups each represented 2.8% of the studies (*n* = 1).

### 3.7. Impact of Emotions on Patient Education Outcomes

**Figure 6:**
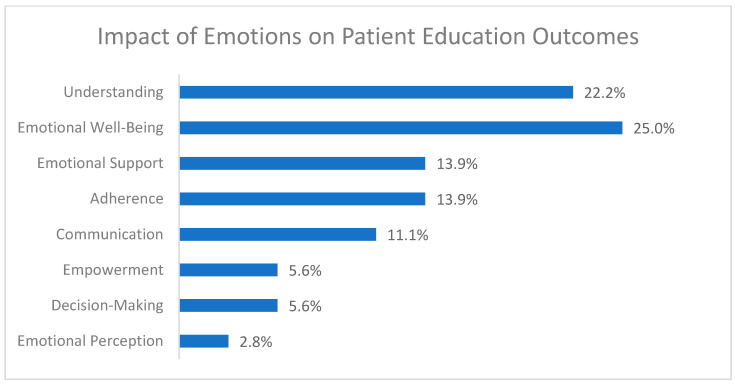
Distribution of patient education outcomes associated with emotions in Emotional Response studies (*n* = 36).

The included studies reported multiple patient education outcomes associated with emotional aspects. The identified outcome categories included understanding, emotional well-being, emotional support, adherence, communication, empowerment, decision-making, and emotional perception. The distribution of these outcomes is presented in Figure 6.

Emotional well-being accounted for 25.0% of the reported outcomes (*n* = 9), followed by understanding with 22.2% (*n* = 8). Emotional support and adherence each represented 13.9% of the outcomes (*n* = 5), while communication accounted for 11.1% (*n* = 4). Empowerment and decision-making each represented 5.6% (*n* = 2), and emotional perception accounted for 2.8% of the outcomes (*n* = 1).

## 4. Discussion

This scoping review synthesized the available evidence on the role of emotions in health literacy and patient education. The findings provide insights into the emotional outcomes most frequently investigated, the methods used to assess emotions, the technologies employed to support patient education, and the influence of emotions on educational outcomes. The following sections discuss these findings in relation to the existing literature and highlight implications for future research and practice.

### 4.1. Emotions in Patient Education

The findings of this review indicate that anxiety and emotional distress were the most frequently investigated emotional outcomes in patient education studies. These results suggest that research has primarily focused on understanding and mitigating negative emotional states associated with illness, diagnosis, treatment, and long-term disease management. This tendency is not surprising, given that many of the health conditions identified in the reviewed studies, particularly cancer, diabetes, and kidney disease, are often accompanied by uncertainty, fear, and concerns regarding prognosis and treatment outcomes.

The predominance of anxiety and emotional distress is consistent with previous evidence indicating that emotional factors can influence how patients process, interpret, and use health information. Emotional responses may affect patients’ ability to understand educational materials, participate in healthcare decisions, and adhere to recommended treatments. Consequently, addressing these emotional dimensions has become an important component of patient-centered education strategies. In this regard, ref. [1] found that lower levels of health literacy were significantly associated with lower levels of happiness among primary-care patients, suggesting that the ability to understand and use health information may influence not only health outcomes but also broader dimensions of subjective well-being.

Similarly, ref. [147] reported that patients with multiple sclerosis frequently experienced anxiety, fear, stress, and emotional distress while searching for health information online. Although most participants demonstrated sufficient e-health literacy levels, concerns regarding the credibility of online information and difficulties in evaluating digital resources remained common. These findings suggest that limitations in health literacy may contribute to negative emotional experiences during health information seeking, reinforcing the importance of educational interventions that address both informational and emotional needs.

Although less frequently reported, other emotional outcomes such as emotional support, reassurance, emotional engagement, and emotional well-being were also identified across the reviewed studies. These findings suggest that patient education is not limited to information transfer but may also contribute to emotional adaptation, confidence building, and coping processes. Furthermore, the association between health literacy and happiness reported by [1] highlights the potential role of patient education in fostering positive emotional states through improved understanding, autonomy, and perceived control over health-related decisions.

However, the relatively low representation of positive emotional outcomes indicates that the literature has largely emphasized the reduction in negative emotions rather than the promotion of positive emotional experiences during patient education. Future research should therefore explore a broader range of emotional outcomes, including happiness, well-being, empowerment, and resilience, to better understand the full emotional impact of educational interventions.

Overall, the results highlight the close relationship between emotional factors and patient education processes. Understanding how emotions influence patients’ experiences may contribute to the design of more effective educational interventions capable of addressing both informational and emotional needs while promoting psychological well-being and quality of life.

### 4.2. Influence of Emotions on Educational Outcomes

The analysis of educational outcomes revealed that emotional well-being and understanding were the most frequently reported effects associated with emotions in patient education. This finding suggests that emotional factors influence not only how patients feel during educational processes but also how effectively they comprehend and retain health-related information. Educational interventions that addressed emotional needs were frequently associated with reduced anxiety, decreased emotional distress, and improved understanding of medical information.

To overcome cognitive and emotional barriers, patient education should move beyond the simple provision of standardized information and adopt more individualized approaches. Evidence from rheumatology suggests that educational needs vary according to patient characteristics, including gender, disease duration, and disease activity levels [144]. Understanding how these factors influence educational requirements may help identify critical therapeutic intervention points during the patient journey, where individuals are more receptive to educational support. Delivering targeted education at these moments may improve treatment adherence, facilitate disease self-management, and help address the emotional burden associated with disease progression and symptom exacerbation. These findings reinforce the importance of developing patient-centered educational strategies that integrate both cognitive and emotional dimensions of healthcare experiences [148].

The strong presence of understanding as an educational outcome highlights the close relationship between emotional states and learning processes. Patients experiencing lower levels of anxiety, fear, or uncertainty may be better positioned to process complex health information, ask questions, and actively engage in educational activities. Conversely, emotional barriers can hinder information processing and reduce the effectiveness of educational interventions. Similar findings have been reported in the health literacy literature, where emotional factors have been shown to influence individuals’ ability to access, understand, and apply health information effectively [1,3].

Adherence and emotional support also emerged as important outcomes. Several studies reported that educational strategies addressing emotional concerns contributed to greater participation in educational programs, improved treatment adherence, and increased engagement with healthcare recommendations. Emotional support was frequently linked to improved coping, resilience, and adaptation to chronic health conditions, reinforcing the importance of considering emotional needs as part of patient-centered educational approaches. Likewise, previous studies have shown that emotional well-being and health literacy are associated with greater self-efficacy and engagement in health-related behaviors [1,149].

In contrast, decision-making and empowerment were less frequently reported outcomes. Although emotional factors can influence healthcare decisions, relatively few studies explicitly examined their impact on decision-making processes. Similarly, empowerment was addressed in a limited number of studies despite its recognized importance in promoting patient autonomy and active participation in healthcare. These findings suggest that future research could further explore the relationship between emotions, patient empowerment, and healthcare decision-making. This gap is particularly relevant given evidence that emotional barriers may influence health-seeking behaviors and participation in healthcare processes [3,4].

Overall, the reviewed studies indicate that emotions influence multiple dimensions of patient education, extending beyond emotional well-being to include understanding, adherence, support, and engagement. This multidimensional impact highlights the need for educational interventions that address both cognitive and emotional aspects of the patient experience. Future patient education initiatives should therefore consider emotional outcomes not only as complementary factors but also as essential components that may shape learning effectiveness, treatment adherence, and patient participation in healthcare decisions.

### 4.3. Technologies Supporting Emotional Aspects of Patient Education

The findings of this review indicate that patient education interventions addressing emotional aspects continue to rely predominantly on traditional educational approaches. Educational programs and educational materials represented more than half of the technologies identified within the Emotional Response studies, while audiovisual resources, counseling-based interventions, and communication strategies were used less frequently. These results suggest that structured educational content remains the primary mechanism for supporting patients’ informational and emotional needs.

The prominence of educational programs and materials may be explained by their accessibility, ease of implementation, and adaptability across different healthcare contexts. Such approaches allow healthcare providers to deliver standardized information while simultaneously addressing concerns related to treatment, disease management, and emotional adaptation. Several studies reported improvements in understanding, emotional support, reassurance, and adherence through the use of these educational resources.

Although digital health solutions were identified, their representation was considerably lower than that of traditional approaches. This finding is noteworthy given the increasing availability of mobile applications, web-based platforms, telehealth services, and other digital tools designed to support patient education. The relatively limited presence of digital health technologies suggests that their potential for addressing emotional dimensions of patient education has not yet been fully explored. Similar concerns have been reported in studies of digital health literacy, which highlight both the opportunities and emotional challenges associated with accessing health information through online environments.

Audiovisual resources, including educational videos and visual materials, constituted another relevant category. These technologies were frequently associated with improved comprehension and emotional engagement, suggesting that visual communication may facilitate the understanding of complex medical information while reducing emotional burden. Similarly, counseling and communication-based interventions highlighted the importance of interpersonal interaction in addressing emotional concerns and supporting patients throughout healthcare processes.

Overall, the results indicate that current patient education practices continue to be dominated by conventional educational approaches, while the integration of more advanced technological solutions remains limited. This observation highlights opportunities for future innovations aimed at combining educational effectiveness with emotional support through technology-enhanced interventions. Recent studies have suggested that emerging technologies such as augmented reality, digital health platforms, and affective computing systems may contribute to more personalized and emotionally responsive patient education experiences [2,6,7].

### 4.4. Assessment of Emotions in Patient Education

The assessment of emotions within patient education contexts was primarily conducted through interviews, surveys, standardized scales, and questionnaires. Interviews represented the most frequently reported method, followed by standardized scales and surveys. This predominance of self-reported and qualitative approaches reflects the traditional reliance on patients’ subjective perceptions to evaluate emotional experiences associated with health information, treatment processes, and educational interventions.

The widespread use of interviews and questionnaires offers several advantages, including ease of administration, low implementation costs, and the ability to capture personal experiences and contextual factors. These methods provide valuable insights into how patients perceive educational materials, cope with health-related challenges, and respond emotionally to healthcare communication. Standardized scales further contribute by enabling more systematic and comparable assessments of emotional states across different patient populations and clinical contexts.

However, the predominance of self-reported measures also presents limitations. Emotional responses are often influenced by memory, self-perception, social desirability, and contextual factors, which may affect the accuracy of reported experiences. Furthermore, these methods typically capture emotions retrospectively rather than during the actual interaction with educational content, limiting their ability to identify dynamic emotional changes in real time.

Another notable finding is the limited use of objective methods for emotion assessment. Few studies incorporated approaches capable of continuously monitoring emotional responses during educational activities. As a result, much of the existing evidence remains dependent on subjective evaluations rather than direct measurements of emotional states. This gap highlights the need for complementary approaches that can provide a more comprehensive understanding of patients’ emotional experiences throughout educational processes. Recent advances in affective computing and emotion recognition technologies have demonstrated the potential of using facial expressions, speech patterns, physiological signals, and multimodal data to identify emotional states more objectively and continuously [2,5].

Overall, the findings suggest that emotion assessment in patient education continues to rely heavily on conventional methodologies. While these approaches remain valuable, future research may benefit from integrating objective and real-time assessment methods capable of capturing emotional responses as they occur during patient education interventions. Such approaches may provide richer insights into the relationship between emotions, learning processes, and patient outcomes [2].

### 4.5. Opportunities for Artificial Intelligence, Emotion Recognition and Augmented Reality

One of the most significant findings of this review is the limited presence of advanced technologies designed to identify, interpret, or adapt to patients’ emotional states during educational interventions. Although digital health solutions were identified in several studies, only a small proportion of the literature was classified within the Artificial Intelligence category, indicating that the integration of intelligent technologies into patient education remains at an early stage.

The findings further revealed that interviews, surveys, questionnaires, and standardized scales continue to dominate emotion assessment practices. While these approaches provide valuable information regarding patients’ perceptions and experiences, they are generally dependent on self-reporting and are often applied after educational interventions have taken place. Consequently, they may not fully capture emotional fluctuations occurring during the learning process itself.

Recent advances in artificial intelligence offer opportunities to address these limitations. Techniques such as computer vision, facial emotion recognition, natural language processing, and multimodal affective computing have demonstrated the potential to detect emotional states in real time and with minimal interruption to user activities [2,5]. The integration of such technologies into patient education systems could enable the continuous monitoring of emotional responses, allowing educational content to be adapted according to patients’ emotional needs, comprehension levels, and engagement patterns.

Similarly, augmented reality represents a promising but largely unexplored area within patient education. Previous research has demonstrated the potential of augmented reality to improve patient understanding, engagement, and satisfaction by providing interactive and visually enriched educational experiences [6,7]. However, few studies have explored how augmented reality environments could be combined with emotion-aware technologies to personalize educational experiences for patients.

The convergence of artificial intelligence, emotion recognition, and augmented reality may create new opportunities for the development of adaptive patient education systems. Such systems could dynamically modify educational content, provide personalized support, and identify emotional barriers that may affect understanding, adherence, and decision-making. By addressing both cognitive and emotional dimensions of learning, these technologies have the potential to enhance the effectiveness of patient education while supporting emotional well-being. Similar opportunities have been highlighted in recent research on healthcare affective computing and intelligent educational systems [2,150].

The available evidence supports identifying these technologies as research opportunities rather than established solutions. The reviewed studies did not provide sufficient comparative evidence to conclude that artificial intelligence, emotion recognition, or augmented reality improves patient-education outcomes. Future studies should therefore evaluate clinical usefulness, accuracy across diverse populations, privacy and informed-consent requirements, algorithmic bias, accessibility, digital literacy, interoperability with clinical workflows, staff training, implementation cost, and the risk of misinterpreting context-dependent emotional signals. Human oversight remains essential, particularly because emotion-recognition outputs should not be treated as direct or definitive measures of a patient’s internal state.

**Figure 7:**
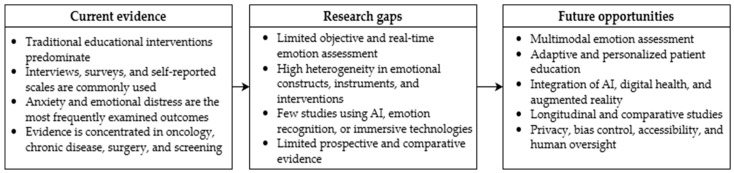
Current evidence, research gaps, and future opportunities for emotion-responsive patient education. The figure summarizes the predominant approaches, the main methodological and technological gaps, and priority directions for future research.

To integrate the main findings of this scoping review, Figure 7 summarizes the current evidence base, the principal research gaps, and priority directions for emotion-responsive patient education.

### 4.6. Limitations

This review has several limitations that should be considered when interpreting the findings. First, the search was limited to Scopus and PubMed, only English-language publications were included, and reference lists were not searched systematically; consequently, relevant studies indexed elsewhere or published in other languages may have been missed. Second, the use of multiple NOT operators improved alignment with the intended scope but may have excluded records containing overlapping terminology. The review also focused specifically on patient education and health literacy, excluding studies centered on mental health, medical education, healthcare professionals, and student populations; therefore, potentially relevant evidence from adjacent healthcare contexts may not have been captured. Third, the included evidence was heterogeneous in health condition, population, educational approach, study design, measurement instrument, and outcome definition, which limits direct comparison across studies. Fourth, many studies relied on interviews, surveys, questionnaires, or other self-reported assessments that are susceptible to recall, social-desirability, and contextual biases. Fifth, methodological quality and risk of bias were not formally appraised because the aim of this scoping review was to map the evidence rather than estimate intervention effectiveness; therefore, the frequency of a reported association should not be interpreted as evidence of its validity, magnitude, or causal direction. Sixth, thematic classification involved reviewer judgment, and some studies could reasonably fit more than one category; assigning one predominant category avoided double counting but simplified multidimensional studies. Finally, country and sample characteristics were not coded uniformly in the original extraction matrix, limiting comparative analysis by geographical setting and population. These limitations mean that the results should be interpreted as a descriptive map of reported research patterns and gaps rather than as evidence that particular emotional or technological interventions are effective.

## 5. Conclusions

This scoping review mapped the role of emotions in health literacy and patient education across 135 included studies. The evidence indicates that emotional factors are frequently examined alongside understanding, adherence, communication, engagement, and emotional well-being. However, because the evidence base is heterogeneous, largely observational, and often based on self-report, these findings describe reported associations and research patterns rather than causal effects.

Among the emotional outcomes identified, anxiety and emotional distress were the most frequently studied, highlighting the strong emphasis placed on addressing negative emotional states associated with illness and treatment. At the same time, positive emotional dimensions such as emotional support, reassurance, emotional engagement, and well-being received comparatively less attention, suggesting important opportunities for future research. The review also revealed that emotional well-being and understanding were the most frequently reported educational outcomes associated with emotions, underscoring the close relationship between emotional and cognitive dimensions of patient education.

The analysis further showed that patient education interventions continue to rely predominantly on traditional educational programs, educational materials, interviews, surveys, and standardized scales. Although digital health technologies were present in the literature, the integration of advanced technologies remained limited. In particular, relatively few studies explored the use of artificial intelligence, emotion recognition, or other objective approaches for assessing emotional responses during educational processes.

This review identified research gaps and emerging technological possibilities. Artificial intelligence, emotion-recognition technologies, and augmented reality may warrant evaluation as components of future patient-education systems, but the current evidence is insufficient to establish their effectiveness, safety, feasibility, or acceptability. Rigorous prospective and comparative studies are needed before such approaches can be recommended for routine practice.

Overall, the evidence indicates that emotions should not be considered secondary factors in patient education. Rather, they represent a fundamental dimension that influences how patients understand, experience, and use health information. Incorporating emotional considerations into the design of educational interventions may contribute to more effective, patient-centered, and responsive healthcare practices.

## Figures and Tables

**Table 1:** Eligibility criteria.

Inclusion Criteria	Exclusion Criteria
Studies related to health literacy, patient education, or health education.	Studies focused primarily on mental health conditions.
Studies addressing emotions or emotional factors in patient populations.	Studies involving students, medical education, nursing education, or healthcare professional training.
Journal articles and review papers.	Studies unrelated to patient education or health literacy.
	Purely technical artificial intelligence studies without a patient education component.
	Editorials, conference papers, book chapters, letters, notes, and short surveys.

**Table 2:** Thematic classification of included studies (*n* = 135).

Category	*n*	%
Artificial Intelligence	5	3.7%
Self-Management	12	8.9%
Patient Empowerment	22	16.3%
Digital Health	23	17.0%
Emotional Response	36	26.7%
Health Literacy	37	27.4%

**Table 3:** Synthesis of the studies included in the Emotional Response category according to the primary emotional outcome (*n* = 36).

Primary Emotional Category	Studies, *n* (%)	Main Health Contexts	Predominant Designs and Instruments	Main Findings
**Anxiety**	11 (30.6%)	Gynecologic surgery; oncology; COPD; kidney disease; childhood asthma; genetic disorders; low-back pain; pregnancy risk communication; type 2 diabetes.	Cross-sectional, qualitative, mixed-methods, intervention, case–control, review, and meta-analytic designs; STAI, HADS, surveys, interviews, and questionnaires.	Lower health literacy, information overload, uncertainty, and unclear risk communication were associated with greater anxiety. Tailored explanations, structured education, and empathetic communication improved understanding and reduced anxiety-related responses.
**Emotional Distress**	10 (27.8%)	Cancer; diabetes; screening; bad-news communication; HIV/AIDS.	Qualitative, mixed-methods, survey, pilot randomized, and professional guidance articles; interviews, PAID, focus groups, vignettes, and content analysis.	Distress was linked to limited understanding, treatment burden, negative educational experiences, and communication difficulties. Personalized education and supportive communication improved coping, knowledge, and participation.
**Fear**	3 (8.3%)	Serious diagnosis; kidney transplantation; critical illness.	Qualitative study, mixed-methods pilot, and professional article; interviews and questionnaires.	Fear arose around serious diagnoses, transplantation, and complex care decisions. Clear information, culturally appropriate education, and supportive communication helped patients process risks without increasing concern.
**Emotional Support**	3 (8.3%)	Cancer survivorship; kidney transplantation; stroke counseling.	Qualitative studies and narrative review; interviews, focus groups, and literature synthesis.	Support from clinicians, family members, peers, and counseling frameworks facilitated access to care, treatment engagement, coping, and decision-making.
**Emotional Perception**	3 (8.3%)	Hypertension; type 2 diabetes; osteoporosis.	Cross-sectional, randomized, and qualitative designs; BIPQ, visual feedback, interviews, and drawings.	Patients’ beliefs and emotional interpretations influenced adherence, risk perception, and understanding. Visual and tailored communication exposed misconceptions and concerns.
**Emotional Engagement**	2 (5.6%)	Safe-sleep education; hypertension management.	Randomized controlled trials; engagement questionnaires and surveys.	Narrative and book-based education increased emotional engagement and was associated with stronger dialogue, adherence, and intentions to change health behaviors.
**Less frequently reported outcomes**	4 (11.1%)	Pediatric cancer; HPV-related cancer; oral cancer treatment; hereditary cancer/genetic information.	Pilot pre–post, qualitative, mixed-methods, and narrative review designs; ratings, interviews, questionnaires, focus groups, and literature review.	Emotional well-being, reassurance, stigma reduction, and general emotional response indicated that concise, culturally tailored, and developmentally appropriate materials can support confidence, coping, and emotional adaptation.

**Note:** Each study was assigned to one primary emotional category. Detailed study-level characteristics are provided in Supplementary Table S2. The studies corresponding to each category are cited in the narrative text following the table.

## Data Availability

The study-level data supporting this scoping review are provided in Supplementary Tables S1 and S2. Table S1 contains the extraction matrix and characteristics of all 135 included studies, whereas Table S2 provides detailed information on the 36 studies classified within the Emotional Response category. No individual-participant data were generated.

## Supplementary Materials

The following supporting information can be downloaded at: https://www.mdpi.com/article/10.3390/healthcare14152424/s1.
